# Gender inequality in cum laude distinctions for PhD students

**DOI:** 10.1038/s41598-023-46375-7

**Published:** 2023-11-29

**Authors:** Thijs Bol

**Affiliations:** 1 Department of Sociology, University of Amsterdam, Amsterdam, The Netherlands; 2 Amsterdam Centre for Inequality Studies, University of Amsterdam, Amsterdam, The Netherlands

**Keywords:** Human behaviour, Scientific data

## Abstract

Resource allocation in academia is highly skewed, and peer evaluation is the main method used to distribute scarce resources. A large literature documents gender inequality in evaluation, and the explanation for this inequality is homophily: male evaluators give more favorable ratings to male candidates. We investigate this by focusing on cum laude distinctions for PhD students in the Netherlands, a distinction that is only awarded to 5 percent of all dissertations and has as its sole goal to distinguish the top from the rest. Using data from over 5000 PhD recipients of a large Dutch university for the period 2011–2021, we find that female PhD students were almost two times less likely to get a cum laude distinction than their male counterparts, even when they had the same doctoral advisor. This gender gap is largest when dissertations are evaluated by all-male committees and decreases as evaluation committees include more female members.

## Introduction

Resource allocation in science is highly skewed^1–3^. Acceptance rates at high-prestige journals are often below ten percent, and the same holds true for prestigious research grants^4^. Consequently, only a small fraction of all researchers can publish in high-prestige journals, receive research grants, or obtain tenured positions. Peer evaluation is key to determining who receives these resources: scholars review each other’s work and determine who belongs to the top and who does not^5^.

While peer evaluation is the dominant method to divide scarce resources, it is not uncontested. A large literature has investigated gender inequality in peer evaluation across a multitude of settings. Many studies find evidence for inequality: journal peer review scores are lower for women^6–8^, men receive higher scores for their grant proposals^9–11^, teaching evaluations are less favorable for women^12–15^, and men are more likely to be evaluated positively in academic hiring^16,17^. The literature is not uniform in this finding, with some recent studies, for example, finding no gender inequality in journal peer review^18,19^ or academic hiring^20^. However, gender inequality in evaluation is argued to be part of the explanation for gender differences in academic careers^21^.

Why do female scholars receive lower evaluations than their male counterparts? Quality could be a first argument, but several studies show that differences remain even when quality is kept constant^11,15,22^. Another important argument lies in the subjective nature of evaluations: evaluations are never objective and cannot be dissociated from evaluators^5,23^. Evaluators’ conception of academic excellence is not neutral but gendered^24,25^, and they are more likely to perceive men as top achievers. Recent studies also find evidence for gender bias in group work, where women receive less credit for their contributions^22^.

Homophily between evaluator and evaluated is also argued to be one of the key reasons for gender inequality in evaluations^8,18,26,27^. Particularly in contexts where there are no neutral or universal criteria that distinguish the top from the rest, evaluators are more favorable about those that are similar to themselves^23^. Since the likelihood to evaluate colleagues (e.g. in hiring committees or grant panels) increases with seniority, the strong overrepresentation of men amongst senior scholars^24,28^ could reproduce existing gender inequalities when male evaluators are more likely to evaluate male academics more positively.

In this article, we study gender inequality in evaluation at the very start of the academic career, by looking at the association between gender and cum laude distinctions for PhD students in the Netherlands. After defending their dissertation, PhD students either obtain a regular doctoral degree or a doctoral degree with a cum laude distinction. Obtaining this distinction is rare: only about 5% of all PhD students are awarded a cum laude. The main doctoral advisor (always a full professor) initiates the procedure to award a cum laude (see Supplementary Appendix for a detailed description of the procedure). A separate dissertation committee consisting of five to seven scholars with relevant expertise then decides. The committee must unanimously agree that the dissertation should be awarded cum laude. If the committee votes for cum laude, two external referees (that are provided by the doctoral advisor) must attest this to be the case. If they all believe the dissertation to rank among the top 5%, a cum laude is awarded. PhD students have no influence on this process and are not informed. Only after defending their dissertation, they will learn whether their dissertation was awarded a cum laude distinction. Candidates who do not receive the award will generally never learn whether a cum laude procedure was initiated.

Some earlier studies investigated gender inequality in doctoral education. Research in Norway showed that men are more likely to enroll in doctorate education^25^. Similar results were found for Germany^29^, but this study showed that academic performance only to a very limited extent explained the gender difference in enrollment. This means that while men were more likely to enroll in doctoral programs, there was no large quality difference between them. A recent study, also in Germany, did show that female doctoral students were slightly less likely to get the summa cum laude recognition for their dissertation^30^.

The empirical case of cum laude distinctions for PhD students provides an interesting context to study gender inequality in evaluations for three reasons. First, there are no clear criteria that define a cum laude distinction—this all depends on what evaluators perceive to be important. This absence of any formal criteria (see Supplementary Appendix for more information) thereby provides a most likely case to observe gender inequality in evaluations. It also allows us to study the role of homophily: most doctoral advisors and members of doctoral committees are male, and we investigate whether male evaluators are more likely to evaluate male PhD students as excellent. Second, since most doctoral advisors have supervised multiple PhD students over the period of observation (2010–2021), we can exploit fixed effects models^31^ where we study gender differences in the likelihood of obtaining cum laude or homophily effects of the dissertation committee within the same doctoral advisor. This allows us to cancel out explanations that point to between-advisor variation, for example in supervision quality, or their likelihood to apply for cum laude. Finally, the only purpose of a cum laude is to separate the exceptional from the rest. This makes the awarding of cum laude distinctions one of the clearest manifestations of the search for excellence that permeates academia^32,33^.

For our analyses, we rely on the information from 5239 PhD students who received their doctorate between 2011 and 2021 at a Dutch public university that is one of the largest research universities in Europe. All PhD students who have defended their dissertations in the mentioned period are part of the sample and were analyzed with data from a university-wide population database. The university under study covers all scientific domains and disciplines. More information on the data collection and processing can be found in the Supplementary Appendix.

## Results

**Figure 1:**
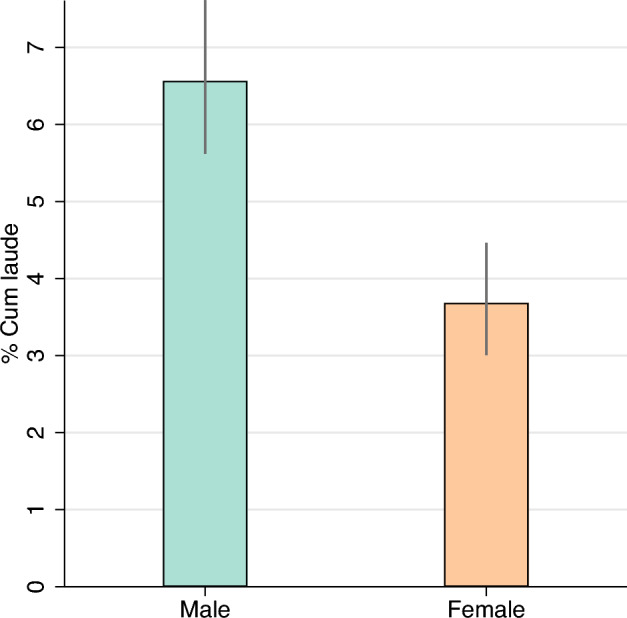
Gender gap in cum laude distinctions. The average gender difference in awarded cum laude distinctions for PhD students. The grand mean by gender for the full dataset. The whiskers represent a 95% exact confidence interval. The difference is significant (*χ*^2^ test, *p* < 0.001, N = 5239).

The data reveal large gender inequality in the probability to obtain a doctoral degree with a cum laude distinction. Figure 1 shows that 6.57% of all male PhD students obtained cum laude, compared to 3.68% of all female students. Compared to dissertations of female PhD students, dissertations of male PhD students are 1.8 times (*χ*^2^ test, *p* < 0.001, N = 5239) more likely to be considered as belonging to the top 5%.

This large gender inequality in cum laude distinctions does not necessarily means that male and female PhD students are evaluated differently. Evaluation practices and definitions of quality vary strongly across scientific domains^23,32^. The observed inequality in Fig. 1 therefore could also be explained by a Simpson’s paradox^34^: female scholars are more likely to obtain their PhD in academic fields where cum laude distinctions are rarely given. Earlier research has for example shown that gender inequality in grant funding^35^ or citations^36^ can be explained by such a paradox.

**Figure 2:**
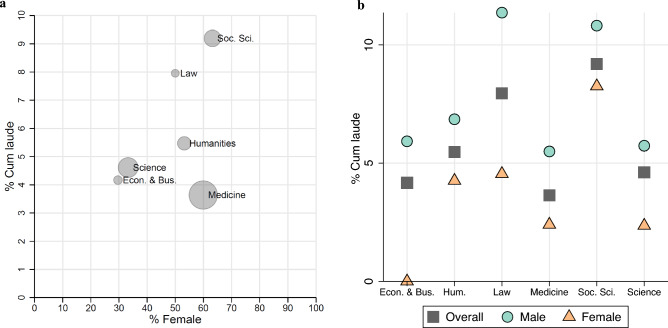
Cum laude distinctions across academic fields. The relation between the academic field and cum laude distinctions of PhD students. The left panel shows the relation between the percentage of cum laude distinctions (y-axis) and the percentage of female PhD students (x-axis) for six academic fields (N = 5239). The size of the markers corresponds to the relative size of that field. The right panel depicts the gender gap in cum laude distinctions for each of the six fields (N = 5239).

Figure 2 evaluates whether differences across academic fields can explain the observed gender gap in cum laude distinctions. The left panel shows the association between the proportion of female PhD students and the likelihood to get a cum laude distinction across different academic fields. A systematic pattern is absent. In Medicine, the probability to receive a cum laude distinction is comparatively lowest (3.6%), while it is highest in the Social Sciences (9.2%). In both fields most PhD students are female. If anything, it seems that cum laude is more often awarded in academic fields with more female PhD students. If the gender inequality in Fig. 1 was explained by a Simpson’s paradox, we would expect the exact opposite. Figure 2 (right panel) shows that the gender gap in cum laude is apparent across all six academic domains. In line with earlier studies^18^, one might hypothesize that the female disadvantage in cum laude would be largest in the academic fields that include the least female academics (i.e. science, economics). While the magnitude of the gap differs, these cross-field differences are not statistically significant (*p* > 0.05).

**Figure 3:**
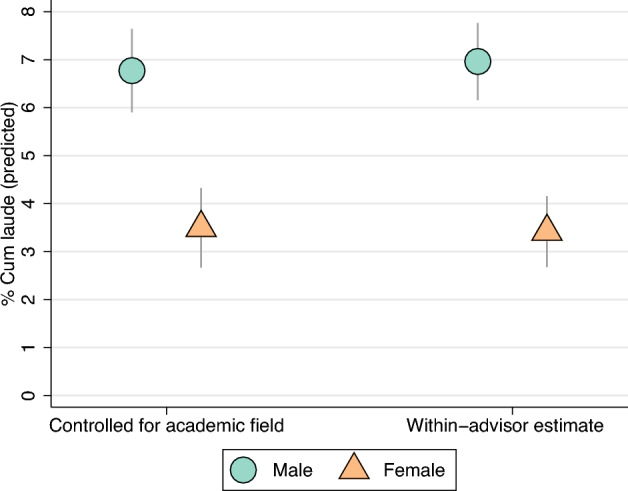
Estimates from regression models. The figure depicts the predicted gap from two regression analyses: (**a**) the predicted gap between male and female PhD students after taking academic field into account (‘Controlled for academic field’), and (**b**) the predicted gap from advisor fixed effects models. For each estimate a normal 95% confidence interval is displayed. The effect of ‘Controlled for academic field’ is significant (*p* < 0.001, N = 5239). The ‘Within-advisor estimate’ is significant too (*p* < 0.001, N = 7249). More information can be found in the Supplementary Appendix, the underlying estimates can be found in Table S3 in the Supplementary Appendix.

The absence of a Simpson’s paradox is confirmed by regression analyses (Fig. 3, left-most markers) where the male advantage in cum laude distinctions remains stable after controlling for academic field (*p* < 0.001). Differences across academic fields do not explain the large gender disparity observed in Fig. 1.

It might be that the definition of fields used in Fig. 2 is too broad, for example, if there are selection effects into more narrow academic fields^36^ or even doctoral advisors. If the supervisory quality of doctoral advisors or their likelihood to apply for a cum laude correlates with the likelihood to supervise a female PhD student, this might explain the observed gender inequality. Figure 3 (right-most markers) presents the results of a regression analysis that includes a fixed effect^31^ for doctoral advisor (see Supplementary Appendix). This estimate can be interpreted as the average within-advisor gender gap in cum laude distinctions. It indicates whether male and female PhD students that were supervised by the same doctoral advisor had a different probability for a cum laude distinction. In this fixed effects specification, the large gender gap is unchanged (*p* < 0.001). Dissertations of female PhD students are, on average, almost twice less likely to get the mark of excellence than their male counterparts with the same doctoral advisor.

**Figure 4:**
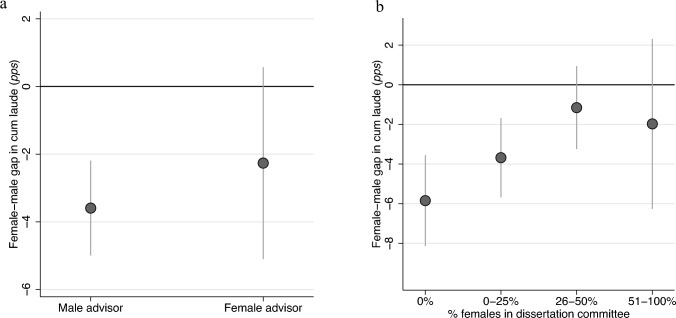
Differences in the gender gap across evaluators. The association between respectively the gender of the doctoral advisors (left panel) and the gender composition of the dissertation committee and the size of the gender gap in cum laude distinctions. The y-axis presents the female-male gap in cum laude distinctions. It indicates how many percentage points (*pp*) female PhD students are less likely to obtain a cum laude distinction. Estimates are obtained from regression analyses that included controls for the academic field and year. For each estimate, a normal 95% confidence interval is displayed. The estimates in the left panel are not significantly different from each other (*p* = 0.38, N = 7219). In the right panel, the difference between full male committees (0%) and 26–50% female committees is significant (*p* = 0.003, N = 7219) More information can be found in the Supplementary Appendix, the underlying estimates can be found in Table S4 in the Supplementary Appendix.

To what extent do we observe gender-driven homophily in cum laude distinctions? Fig. 4 evaluates this by looking both at the gender of the doctoral supervisors (left panel) and the gender composition of the committee that evaluates the dissertation (right panel). In both figures the y-axis presents the female-male gap in cum laude distinctions in percentage points (*pp*). For doctoral advisors (Fig. 3, left panel), we find little evidence for gender bias homophily. Female PhD students are less likely than male PhD students to obtain cum laude irrespective of whether their doctoral advisor is male (3.6* pp* less likely) or female (2.3* pp* less likely). While the gender gap in cum laude distinctions is smaller when the advisor is female, this difference between male and female doctoral advisors is not statistically significant (*p* = 0.38).

The dissertation committee plays a crucial role in awarding the cum laude distinction too: they must agree that the dissertation belongs to the top 5%. For the committee, we find a pattern of gender homophily (Fig. 4, right panel). These results are again based on a fixed effects specification and exploit the variation in committee composition *within* the same doctoral advisor. There is no exogenous variation in committees^37^, and doctoral advisors have an important say in determining the composition of the committee. However, the fixed effects approach rules out that the observed pattern of gender homophily stems from variation across doctoral advisors: all effects are estimated within doctoral advisors.

The gender gap in cum laude is largest when the dissertation is evaluated by an all-male committee: 5.8* pp*. In this scenario, 9.1% of male PhD students obtain a cum laude distinction, compared to 3.3% of female PhD students with the same doctoral advisor—a difference by a factor of almost 3 (see Supplementary Appendix Fig. S5). The gender gap in cum laude distinctions decreases as the gender composition shifts towards a greater number of female committee members. For dissertations that are evaluated by a committee where at least a quarter of the members is female, the gender gap in cum laude distinctions disappears. More balanced dissertation committees were a minority. Over the observed period, 9.6% of all dissertations were evaluated by a committee where more than half of the members were female, whereas 27.8% were evaluated by an all-male committee (see Supplementary Appendix Table S1).

We have performed several robustness checks. First, some studies have argued that gender inequality in evaluation is decreasing over time^38^, additional analyses show that the found pattern of gender inequality in cum laude distinctions remains stable over the observed period (2011–2021) (see Supplementary Appendix Fig. S8). Second, the seniority of committee members might explain the homophily results here, given that female committee members might be less likely to be senior. We do not find evidence for this, and the presented results are robust when we include committee members’ seniority (see Supplementary Appendix Table S6). Finally, next to doctoral advisors, PhD students often have co-supervisors (see Supplementary Appendix). They are involved in supervision but do not have the formal right to hand out doctoral degrees. Our finding that there are no homophily effects for doctoral advisors holds when we look at the gender composition of the full supervisory team (see Supplementary Appendix Fig. S9).

## Discussion

We conclude that there is substantial inequality in the extent to which the work of early researchers is evaluated and perceived as excellent. Male PhD students are almost twice as likely than female PhD students to obtain a cum laude distinction. The gender of the doctoral advisors is not significantly associated with the size of the gap, but the gender composition of the committee is: the average gender gap in cum laude distinctions is largest for all-male committees and nears zero when committees are getting closer to gender parity.

A limitation of the current analysis is that we do not know which committee member has voted for or against cum laude, which means that we were only able to estimate homophily effects in the composition of the evaluation team. Moreover, we do not have any information on the two external anonymous reviewers that also have to attest that the dissertation deserves cum laude. Although the names of these external referees are provided by the doctoral advisor(s), we cannot rule out that they also contribute to the observed gender inequality in cum laude distinctions. Earlier studies also pointed to the unequal effects of parenthood to understand gender inequality in academia^39^. While we do not have data on parenthood, only a small fraction of PhD students become parents during the writing of their dissertation^40,41^. In this study, we were also not able to investigate whether other aspects of the work relationship between PhD student and doctoral advisor (e.g. if they taught together) plays a role. A final limitation of the current study is that it is based on one university. While it might be that gender inequality differs across universities in the Netherlands, the studied university is broad and not limited to a few academic fields. Moreover, recently, a newspaper reported gender inequality in cum laude across most Dutch universities^42^.

A puzzling finding is that there are homophily effects for the committee but not for the doctoral advisors. The data do not allow us to explore this finding further. A possible explanation might be that supervisors build stronger relationships with their students than committee members, which makes their assessment more accurate.

Most prominently, with the current data we are unable to fully rule out that the observed gender inequality in cum laude distinctions is explained by something else than gender bias^43^. It might for example be that male PhD students are overrepresented in the right tail of the “quality distribution”. Existing research provides little evidence for this. Among school-aged children, both boys and girls are overrepresented in the right tail depending on which skill is tested (numeracy or literacy)^44,45^. To isolate the part of the effect that is driven by bias would be to “control” for the quality of the PhD dissertation (e.g. publications, citations for dissertational work). However, this will not help us further since these measures of quality are themselves affected by gender bias^21,43,46^.

Another alternative explanation for our findings might be found in a gender gap in competitiveness^47,48^. PhD students are not aware that their doctoral advisors apply for cum laude and have no influence over this decision. It is therefore highly unlikely that the gender gap in cum laude distinctions is driven by differences in strategic choices concerning the application process^49^. Moreover, the reported gender gaps in competitiveness are much smaller than the effects found.

While we are unable to interpret the observed gender inequality as bias, we do believe that our results provide strong indications for a biased perception of excellence. The gender gap in cum laude is not explained by differences across academic fields or doctoral advisors: within the same doctoral advisors, male PhD students are almost two times more likely to obtain a cum laude. Nevertheless, only in a controlled experiment where quality is kept constant, we would be able to identify the causal effect of gender on cum laude distinctions. This is not possible in the current study, and its main contribution was to document the association between gender and the cum laude distinction. Future studies should try to triangulate the finding in more controlled designs.

How big is the career advantage for those who obtain a cum laude distinction? In other words, how important is it? While it is difficult to quantify this, graduating cum laude is an official criterion of the largest Dutch grant funder to signal the quality of the researcher. More generally, it is used as a mark of excellence, particularly in job applications for early career researchers. We believe that the findings of this study bear relevance besides the importance of cum laude, as the current study shows that already in the early career there is a large gender inequality in who is perceived as excellent. Men are more often deemed to belong to the top than women, particularly when evaluated by men.

Recent studies indicate that gender inequality in academia is pervasive^21,50^. This study is no exception. Cum laude distinctions might be functional for those who obtain them, as they obtain the benefits associated with them. At the same time, the cum laude distinction is an instrument that leads to inequalities that are unlikely meritocratic in nature. In line with earlier work^49^, this research indicates that gender inequality in academia is at least to some extent driven by institutional barriers that tend to be higher for women than for men. Obtaining a cum laude distinction is such a barrier, that is easier to pass for men than for women.

How to solve this inequality? Double-blind reviewing is seen as one way to mitigate gender inequality^51^, but in the context of evaluating dissertations, this will be difficult as by definition the majority of committee members work at the same university and is likely to know the PhD student. The current study presents evidence for gender bias homophily in evaluators. A straightforward solution would therefore be to enhance the gender balance in evaluation committees in academia. However, given the gender segregation across academic fields (see Fig. 2, left panel), for some academic disciplines this will be unachievable in the short term—or it would put even greater pressure on the small group of female academics in those fields.

We believe that this study raises a more fundamental question: is it always crucial to distinguish the excellent from the rest? In some cases, such as hiring, it is unavoidable: there are not enough jobs for everyone. But academia is rife with prizes, awards, and distinctions, that often serve no other purpose than marking some as excellent. We can question whether these institutional barriers provide large functional benefits for academia, but the current study does show that they can perpetuate existing inequalities. Debates about bias in the evaluation of excellence therefore should not just be about how to create equal opportunities when men and women face institutional barriers in academia, but also about the necessity of institutional barriers in the first place.

## Materials and methods

### Data

In this study, we analyze information from one of the largest public universities in The Netherlands. The data contained information on all PhD students that completed their dissertations at the university between 2011 and 2021. In total, 5240 dissertations were written over this period. All dissertations are part of the data, which means that we analyze population data for the university.

The university under study is one of the largest research universities in Europe. It employs over 3000 researchers and is home to 40,000 students. The university is organized into seven faculties that cover the breadth of academic research: Medicine, Law, Social Sciences (e.g. Sociology, Psychology), Humanities (e.g. Philosophy, Language, History), Economics and Business, and Science (e.g. Physics, Mathematics, Biology). All large academic disciplines are represented in the university, with engineering as the exception. In the Netherlands, Engineering Schools can be found in the three technical universities, and hence PhD dissertations written in the technical domain were not part of the data.

The data consists of 5239 dissertations—one observation is set to missing because no doctoral advisors were registered. PhD students can have multiple doctoral advisors, and many doctoral advisors have supervised multiple PhD students in the observed period (2011–2021) (see Fig. S4 in the Supplementary Appendix).

In our data, we identified 1623 unique doctoral advisors *j*, that have supervised a total of 5239 PhD students *i*. In total, there were 7249 doctoral advisor–PhD student combinations *ij* in our data. In the regression analyses, we analyzed different samples, depending on the method or research question. For example, we analyzed the sample of PhD students (N=5239) when we were interested in variation between PhD students (for example in calculating the raw difference in cum laude distinctions between men and women), but the sample of all PhD student–doctoral advisor combinations (N=7249) were analyzed when we were interested in the role of the doctoral advisors or committee.

We have collected different information on the dissertation and the PhD student from the university databases. First, for each dissertation we knew the gender of the PhD student, the date of obtaining the PhD, the broad academic field the dissertation was written in, and whether the dissertation was awarded cum laude. Second, for the supervisors, we knew their roles in the supervision (doctoral advisor or co-supervisor), names, gender, and academic title. This latter information allows us to separate full professors from assistant and associate professors, as only the first is allowed to carry the “Prof.” title. The academic title thereby is a good proxy of the seniority of the doctoral advisor (or co-supervisor). Finally, the data contained information on the dissertation committee. For them, we have collected their gender and academic title. Table S1 in the Supplementary Appendix provides an overview of all measures used in the study.

### Statistical analyses

Some results in the article are descriptive, but others are obtained by performing different types of regression analyses. For all analyses, we estimated a linear probability model^52,53^ on our binary dependent variable *y*_*i*_, which measures whether the dissertation of PhD student *i* did (1) or did not (0) receive a cum laude distinction. While we use linear probability models, logistic regressions provide the same findings (see Supplementary Appendix Fig. S10). We prefer linear probability models over logistic regression because several studies have highlighted difficulties with calculating standard errors and interpreting interaction effects in logit (and probit) models^54,55^. To obtain the results presented in the article, we use three different regression models, depending on whether we analyze the sample of PhD students *i* or the sample of all combinations between PhD students *i* and doctoral advisors *j*.

First, we have used a simple linear regression at the level of PhD students (N=5239) to estimate the results in Fig. 1 and the left estimates in Fig. 3. Here we regressed obtaining cum laude on the gender of the PhD student. In this model, we could add several controls measured at the level of the PhD student (year of obtained PhD; academic field). Details on the model and the estimated results can be found in the Supplementary Appendix.

Second, we have estimated multilevel regressions on the sample of all combinations between PhD students (*i*) and doctoral advisors (*j*) (N=7239). We used these models because we wanted to know whether gender inequality in cum laude distinctions differed across male and female doctoral advisors (Fig. 4, left panel). The data structure is cross classified: doctoral advisors had multiple PhD students, but PhD students also had multiple doctoral advisors. To correct for this we cluster standard errors within PhD students. Details on the equation and the estimated results can be found in the Supplementary Appendix.

Finally, we used the combined sample of doctoral advisors (N=7239) to estimate fixed effects models. By including a fixed effect for the doctoral advisor *j*, all between-doctoral advisor variance is accounted for, and the only variance that is left to explain was that within each advisor. This means that we estimated whether, on average, the same doctoral advisor was more likely to have male than female PhD students with cum laude distinctions (Fig. 3, right estimate). In a similar way, we exploited the variation in the gender composition of the dissertation committees within the same doctoral advisors and estimated whether, on average, for the same supervisors the gender inequality in cum laude depended on the percentage of female members of the dissertation committee (Fig. 4, right panel). An important benefit of these models is that we were able to rule out the variation between doctoral advisors as a source of variation for gender inequality in cum laude. More information on the fixed effects models and their assumptions, as well as the obtained results, can be found in the Supplementary Appendix.

The current study and data collection has been approved by the Ethical Committee of the Amsterdam Institute for Social Science Research of the University of Amsterdam. All methods were carried out in accordance with the guidelines and regulations of the University of Amsterdam. The need for informed consent was waived by the Ethical Committee of the Amsterdam Institute for Social Science Research of the University of Amsterdam.

### Supplementary Information

Supplementary Information.

## Data Availability

All code and data are available via https://osf.io/3fqe2/. The data are only available in anonymized form. Full data access (including identifiable information) will only be possible when granted by the supplier of the university that was studied.
