# Supplementary material for: Gender inequality in cum laude distinctions for PhD students
Source: Sci Rep. 2023 Nov 29;13:20267. doi: 10.1038/s41598-023-46375-7 (PMC10687083; doi:10.1038/s41598-023-46375-7)
Supplement: Supplementary file 1 — Supplementary Information. [file 41598_2023_46375_MOESM1_ESM.pdf]

Supplementary Appendix for

Gender inequality in ‘cum laude’ distinctions for PhD students

Thijs Bol

Contents

1 Description of 'cum laude' application **3**

1.1 Description of PhD trajectory in the Netherlands 3

1.1.1 Supervisors 3

1.1.2 Dissertation committee 4

1.1.3 Cum laude procedure 6

2 Data and methods **7**

2.1 Measures 8

2.2 Methods 10

3 Results from regression models **13**

3.1 Main effect of gender of PhD 13

3.2 Interaction analyses with advisors and dissertation committee 14

4 Robustness checks **18**

4.1 Trend over time 18

4.2 Role of co-supervisors 19

4.3 Seniority of committee 20

4.4 Logistic regressions 22

1 Description of 'cum laude' application

1.1 Description of PhD trajectory in the Netherlands

In the Dutch system, PhD students are paid employees that get paid for three to four years to finish their dissertation. Depending on the academic field the PhD student is in, they can obtain a doctoral degree by writing journal articles or a monograph, where the latter is most common in the Humanities and some parts of the Social Sciences. PhD trajectories normally take 3 or 4 years, although delays do happen.

In the following we will describe the formal characteristics from the start of a PhD project to obtaining the doctoral degree, as laid out the doctoral regulations for the university under study (see Section 2 for more info on the specific case). Across universities in the Netherlands there is some variation in doctoral regulations, although the process is generally very similar to the one described here.

1.1.1 Supervisors

In the supervisory team of the PhD student supervisors can take two roles: the doctoral advisor, and the co-supervisor (also known as the "co-promotor"). All PhD students have at least one doctoral advisor, as they are the only ones with the *ius promovendi*, the legal right to act as main doctoral advisor (the "promotor") and award PhD students a doctoral degree. The doctoral board of the university will only allow a PhD student to enter the doctoral program when there is at least one doctoral advisor with the *ius promovendi*.

In the period under study, the right to act as doctoral advisor was only given to full professors.[1] PhD students can have multiple doctoral advisors, although it rarely happens that there are more than two doctoral advisors. Fig S1a shows that in our data the majority (62%) of PhD students had only one doctoral advisor. 38% had two doctoral advisors, and in 5 cases (0.1%) there were three full professors acting as doctoral advisor.

Besides their doctoral advisor, co-supervisors are often also part of the supervisory team of a PhD student. Co-supervisors are not obliged to have the *ius promovendi*, and they do not have to be a full professor. To the contrast, very often co-supervisors are assistant or associate professors. The only requirement that the doctoral board of the university has for co-supervisors is that they possess a doctorate themselves. In our data, the total supervisory team (doctoral advisor(s) + co-supervisor(s)) consisted of 2-4 people (see Figure S1b). 8% of all dissertations was supervised by only a doctoral

[1] In the past two years, associate professors can also get the *ius promovendi* when certain conditions that are specific to universities and/or faculties are met.

Figure S1: Supervision of PhD students

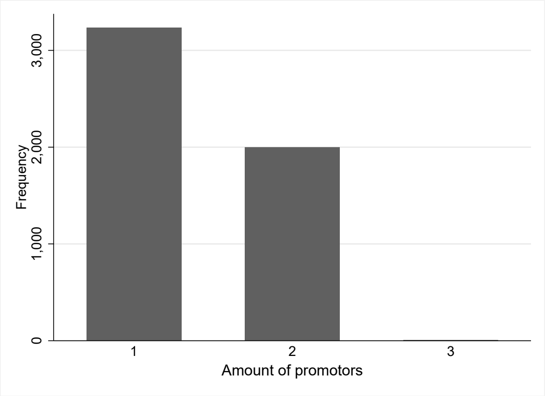

(a) Total number of doctoral advisors

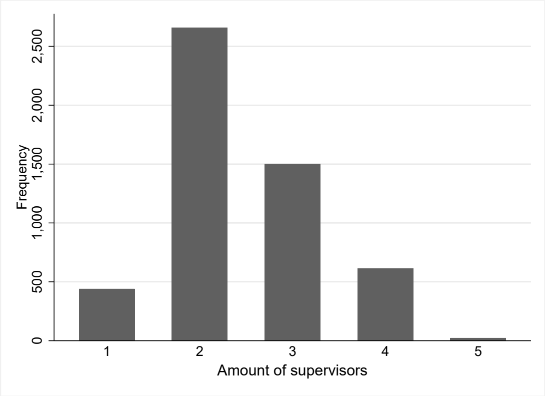

(b) Total number of supervisors

advisor. The Doctoral Board of the University has to approve of the full supervisory team, although this is a formality when the above-mentioned conditions are met.

1.1.2 Dissertation committee

When the PhD thesis is almost finished, the supervisory team submits a proposal for the composition of the dissertation committee that will evaluate the dissertation and decide whether the candidate deserves the doctoral degree. The dissertation committee normally consists of at least five and at most seven members that all have voting rights about awarding the doctorate. Figure S2 shows that in our data the dissertation committees almost always consisted of 5-7 members, although a small fraction of the dissertation had committees with less than 5 members (3.5%) or more than 7 members (0.2%).

The supervisory team has the discretionary space to propose members of the dissertation committee, but there are some requirements. The majority of the voting members must be full professor, and at least half of them must be affiliated to the university where the doctoral degree is obtained. At least one member of the dissertation committee has to be a full professor working in the same faculty as where the PhD will get the doctorate, and at least one member must be from another (foreign) university. There are no rules with respect to the demographic composition (e.g., gender) of the dissertation committee.

When assessing the dissertation, committee members are asked to pay attention to several factors, including the clarity and relevance of the research puzzle, the robustness of the methods, the structure of the thesis and the extent to which the thesis is innovative. At least eight weeks before the defence ceremony, the committee members submit their vote for or against admitting the PhD student to the PhD ceremony. This vote cannot be conditional upon revisions in the dissertation. In contrast to many other countries, dissertation committees in the Netherlands face

Figure S2: Committee members

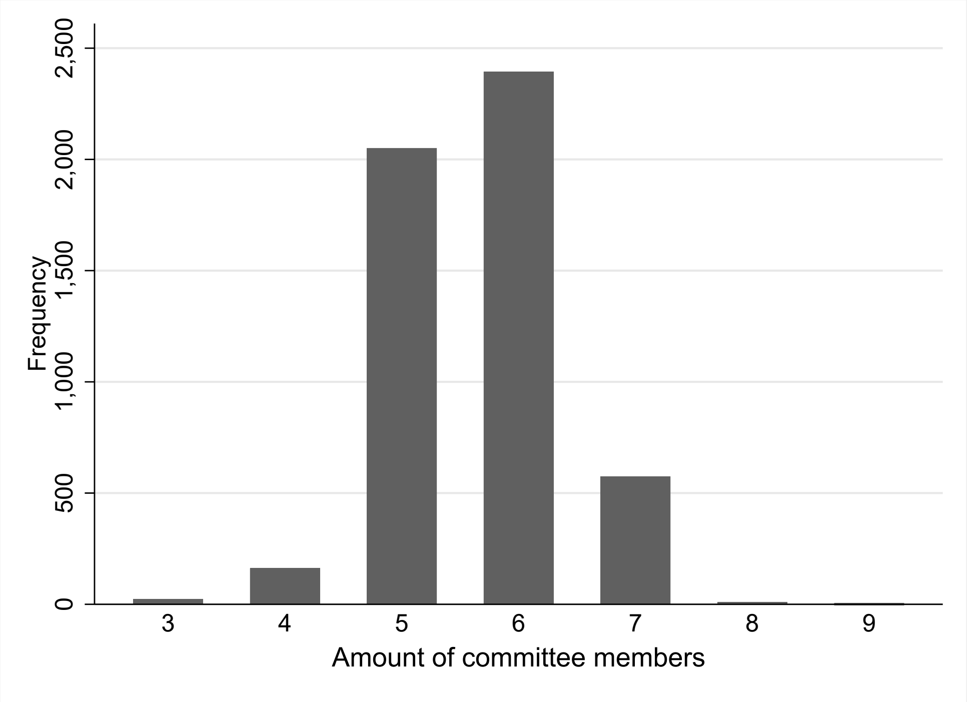

a binary decision: either they approve the dissertation or they do not. This means that there is no room for small adjustments or revisions, and the dissertation as handed in has to be approved or rejected. As an outcome, a vote against admitting the dissertation to the defence ceremony only happens when a committee member observes (a) major flaw(s) in the manuscript. Admission to the PhD ceremony is decided by a majority vote.

At the defence ceremony, the PhD student engages in a discussion with all members of the dissertation committee. The defence takes exactly 45 minutes, and in this time the committee members get the opportunity to ask questions and voice concerns, whereas the PhD student gets the opportunity to defend his/her/their thesis. The ceremony is public, and everyone interested is allowed to join.

After the defence, the committee retires to discuss the defence and dissertation. This deliberation takes place behind closed doors with only the doctoral advisor(s), co-supervisors, and dissertation committee members present.[2] First, the doctoral advisor(s) and co-supervisor(s) advise to the committee whether they believe the candidate should be awarded the doctorate and then all committee members vote for or against awarding the doctoral degree. This again is decided by a majority vote. Since the decision to allow the candidate to the ceremony has already been taken at this point, it is extremely rare that the doctoral degree will not be awarded at the ceremony. It is so rare that it has not happened at the university that we analyzed for

[2]There is also a chair of the promotion committee, but the chair has no voting rights and has presiding the defence and the deliberation afterwards as its main task.

the period under study (2011-2021). When the committee has made its decision, they return to the room of the public defense and award the PhD student with the doctoral degree.

1.1.3 Cum laude procedure

In this article we are mostly interested in ’cum laude’ distinctions, but how does the awarding of a ’cum laude’ work? In order to award dissertations there are several steps that need to be taken, and we will discuss all of them chronologically. A schematic overview of the full procedure can be found in Figure S3.

Figure S3: Schematic overview of cum laude procedure

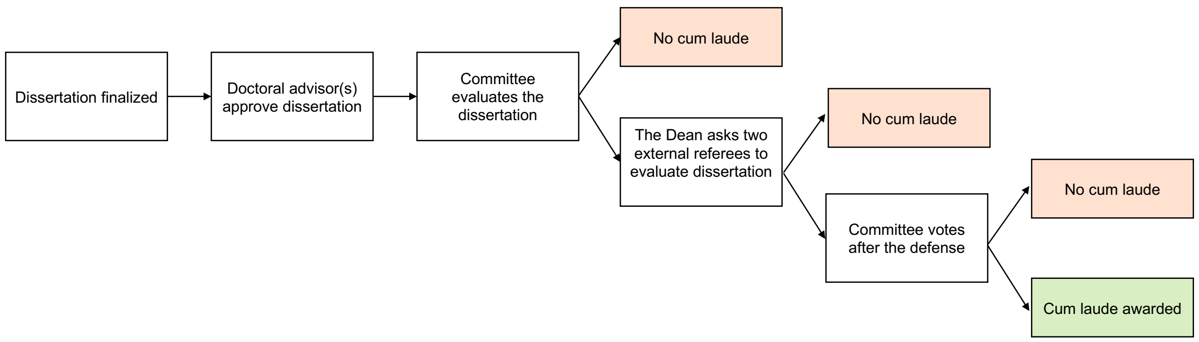

After finalizing the dissertation, and the doctoral advisors have approved of the dissertation, it is sent out to the dissertation committee, who will review the dissertation. After their evaluation of the dissertation, each committee member has to answer two questions related to ’cum laude’: (a) whether they believe that this doctoral thesis should qualify for the distinction ‘cum laude’?, and (b) whether they can attest that the dissertation belongs to the top 5% of all dissertations that this committee member read in the relevant field of study. Both questions can only be answered with a simple yes or no.

When the assessments of the committee members are in, the doctoral advisor(s) can propose a ’cum laude’ to the Dean of their Faculty. Such a ’cum laude’ procedure can only be started when no more than one committee member answered "no" to (one of) the aforementioned two questions. If this criteria is met, the doctoral advisor has to write a detailed and reasoned proposal to the Dean explaining why ’cum laude’ is merited. In that proposal, the doctoral advisor has to name at least four independent external referees. These referees cannot be member of the dissertation committee and the list of external referees has to include scholars working at foreign universities.

The Dean will, without consulting the doctoral advisor(s), choose two of the external referees from the list. These external referees remain anonymous to the doctoral advisor(s) and the PhD student. The two external referees read the dissertation and submit a motivated recommendation explaining whether or not the dissertation de-

serves ’cum laude’. Both external referees have to agree that the dissertation belongs to the top 5% of that respective field: if one external referee gives a negative advice, the ’cum laude’ is off the table.

If both external referees give a positive advice, the dissertation committee has the deciding vote. However, only after the PhD defence they will vote whether the dissertation receives ’cum laude’. This process takes place behind closed doors, at the moment that the dissertation is deliberated by the doctoral advisor(s), co-supervisor(s), and committee members. Before the defence starts, the university prepares two different doctoral degrees: one with, and one without ’cum laude’ on it. After the defence, the doctoral advisor(s) and co-supervisor(s) advice the committee on whether they believe the dissertation deserves ’cum laude’. The dissertation committee members then anonymously vote in writing. After all votes are in, they are counted by the chair of the dissertation committee. When no more than one vote is against, the PhD student receives the doctoral degree with ’cum laude’ on it. The other doctoral degree, without ’cum laude’ on it, is torn apart and thrown away.

When the discussion behind closed doors is ended, the dissertation committee and supervisors return to the public room of the defence where the PhD student gets awarded his or her doctoral degree. It is only at this point that the PhD student will learn whether the dissertation receives ’cum laude’. Since the final decision for ’cum laude’ will only be made just before awarding the doctoral degree, PhD students are not being made aware of all the steps that are to taken before. PhD candidates do not know if ’cum laude’ is on the table at the start of the defence, but only will learn afterwards.

2 Data and methods

The data consisted of 5,239 dissertations, all defended between 2011 and 2021.[3] All information that is used in this article is obtained from a central database of the university. In this database, information on the doctoral thesis, PhD student, advisors, and committee is stored at the moment of the defense of the PhD student. The data analyzed in this article were therefore not collected with data analysis as its purpose, they are obtained from the university’s registers. This also means that some relevant information that we would have liked to analyze is unavailable. Staff of the studied university have processed the data and have provided us with the raw data from the university database.

PhD students can have multiple doctoral advisors, and a large number of doctoral advisors have supervised multiple PhD students in the observed period (2011-2021) (see Figure S5). Figure S4 provides a schematic overview of the data. In our data we

[3]One observation is set to missing because no doctoral advisors were registered.

Figure S4: Schematic overview of data

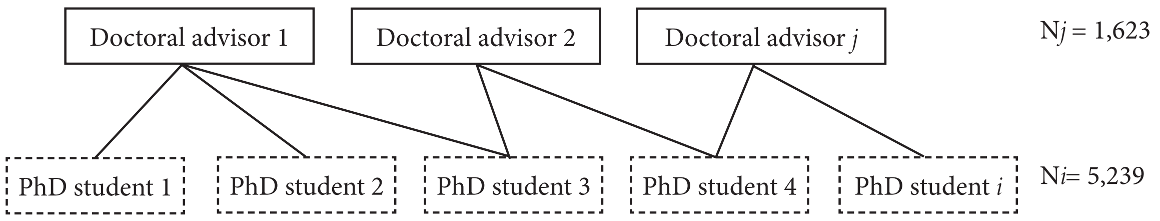

identified 1,623 unique doctoral advisors *j*, that have supervised a total of 5,239 PhD students *i*. In total there were 7,249 doctoral advisor–PhD student combinations *ij* in our data. This means that in the observed period a PhD student had on average 1.38 doctoral advisors. In the regression analyses (see below) we have analyzed different samples, depending on the method or research question. For example, we analyzed the sample of PhD students (N=5,239) when we were interested in variation between PhD students (for example in calculating the raw difference in 'cum laude' distinctions between men and women), but the sample of all PhD student–doctoral advisor combinations (N=7,249) were analyzed when we were interested in the role of the doctoral advisors or committee. For each analysis used in the article we will specify the sample it was estimated on in Section 3.

2.1 Measures

Table S1 provides an overview of all measures used in the study. It is important to note that the sample size is not fully constant across all measures. This is mostly due to the level at which the variables are measured (the level of the doctoral advisor or the level of the PhD student). There are 24 missing observations (0.5%) for the gender composition of the dissertation committee, as for these dissertations the committee was not registered.

Table S1 makes clear that overall, 5% of all dissertations received 'cum laude', and more than half of the PhD students were female. In Table S2 we see that descriptively there are relatively many more male PhD students that have obtained a cum laude distinction compared to female PhD students. This is also visualized in Figure 1 in the main article.

Dissertation committees rarely contained more than 50% females: only in 10% of the dissertations, the majority of the dissertation committee was female. 28% of all PhD students defended their thesis in front of a committee consisting solely of men. The table also makes clear that the amount of PhD dissertations that were written remains quite stable over the observed period. The exceptions here were 2011 and 2021, but this had to do with data availability: data was not available for the full calendar years at the moment of data collection.

Finally, it is important to note that we do not know for each individual committee member whom voted for or against 'cum laude'. As explained above, the voting after the PhD defence takes place anonymously, which makes it impossible to track whether someone voted against. We do know that this can never be more than one committee member, as otherwise the 'cum laude' would not be awarded. Finally, in the process of awarding a 'cum laude', two external referees must also agree that the dissertation belongs to the top 5% of that field. The university does not systematically register the reports of these external referees, and therefore they were not part of the analyses.

Table S1: Description of data

	Mean	Range	SD	N
**PhD student**				
Cum laude (1=yes)	.05	0-1		5,239
Gender PhD (1=female)	.52	0-1		5,239
Academic domain				
Economics and Business	.04	0-1		5,239
Humanities	.10	0-1		5,239
Law	.03	0-1		5,239
Medicine	.45	0-1		5,239
Social Sciences	.15	0-1		5,239
Science	.22	0-1		5,239
# Doctoral advisors	1.38	1-3	.49	5,239
# Total supervisors	2.45	1-5	.82	5,239
Dissertation committee				
0% Female	.28	0-1		5,215
1-25% Female	.32	0-1		5,215
26-50% Female	.31	0-1		5,215
51-100% Female	.10	0-1		5,215
Year of defense				
2011	.07	0-1		5,239
2012	.09	0-1		5,239
2013	.10	0-1		5,239
2014	.09	0-1		5,239
2015	.09	0-1		5,239
2016	.10	0-1		5,239
2017	.10	0-1		5,239
2018	.10	0-1		5,239
2019	.10	0-1		5,239
2020	.10	0-1		5,239
2021	.04	0-1		5,239
**Doctoral advisor**				
Gender (1=female)	.19	0-1		1,628
# of PhD students	4.45	1-58	5.72	1,628

In our data, only one in five of the doctoral advisors was female. Given that the *ius promovendi* was only given to full professors in the observed period, the low percentage of female doctoral advisors is most likely explained by the low number of female full professors in the Netherlands [1]. Finally, Table S1 shows that, on average, doctoral advisors have supervised multiple PhD students in the observed time period. On average, they have supervised 4.45 PhD students, with a maximum of 58. This

variation is important for the fixed effects models, as explained below.

Table S2: Cross table of gender PhD and cum laude

	Male PhD	Female PhD	**Total**
No cum laude	2,334	2,640	**4,974**
Cum laude	164	101	**265**
Total	2,498	2,741	**5,239**

2.2 Methods

We use different regression models, depending on whether we analyze the sample of PhD students i or the sample of all combinations between PhD students i and doctoral advisors j.

The first model is estimated with a simple linear regression where our binary dependent variable y_i, which measures whether the dissertation of PhD student i did (1) or did not (0) receive a 'cum laude' distinction. The basic model in Equation 1 shows that y_i was regressed on X_{1i}, a dummy indicator measuring the gender of the PhD student. The key parameter of interest here was β_1, which provides the predicted effect of gender of the PhD student on obtaining 'cum laude'. The vector ζ_i denotes the effects for different control variables that are added (see Table S3 for all controls that are added), ϵ_i denotes the residual term.

$$y_i = \alpha_i + \beta_1 X_{1i} + \zeta_i + \epsilon_i \tag{1}$$

Second, in order to investigate whether gender inequality in 'cum laude' distinctions differed across male and female doctoral advisors, we estimated multilevel models. Here we used the sample of all combinations between PhD students (i) and doctoral advisors (j). Equation2 depicts the statistical model that was used to answer this question. The key difference with Equation 1 is the presence of term β_2, that denotes the predicted effect of the gender of the doctoral advisor X_{2j}, and the interaction term β_3 between the gender of the doctoral advisor and the gender of the PhD student. The vectors ζ_{ij} and ρ_j include control variables measured at the level of the PhD student or the doctoral advisor. The data structure is cross-classified: doctoral advisors had multiple PhD students, but PhD students also had multiple doctoral advisors. To correct for this we cluster standard errors within PhD students, to get the most robust estimate of the predicted effect.

$$y_{ij} = \alpha_{ij} + \beta_1 X_{1ij} + \beta_2 X_{2j} + \beta_3 X_{1ij} * X_{2j} + \zeta_{ij} + \epsilon_{ij} \tag{2}$$

Third, we estimated fixed effects models. By including a fixed effect for the doctoral advisor j, all between-doctoral advisor variance is fixed, and the only variance that is

left to explain was that within each advisor. Equation 3 presents the basic setup of this fixed effects model. It is similar to Equation 1, but has as the key exception the term δ_j, which depicts a fixed effect for doctoral advisor.

$$y_i j = \alpha_i j + \beta_1 X_{1ij} + \zeta_i j + \delta_j + \epsilon_i j \tag{3}$$

An important benefit of these fixed effects models is that we were able to rule out variation between doctoral advisors as a source of variation for gender inequality in 'cum laude'. In order to be able to estimate such a fixed effects models, it is important to have sufficient variation within doctoral advisors. In other words: the data had to contain enough doctoral advisors that supervised multiple PhD students. Figure S5 shows the frequency of doctoral advisors depending on the number of completed dissertations in the observed period (2011-2021). A bit over 40% of all doctoral advisors have only supervised one finished dissertation in the observed period. For these doctoral advisors there is no within-variance, which means that they did not contribute to the estimated effect of β_{1ij} in Equation 3. At the same time, almost 60% of the doctoral advisors did supervise multiple PhD students.

Figure S5: PhD students per doctoral advisors

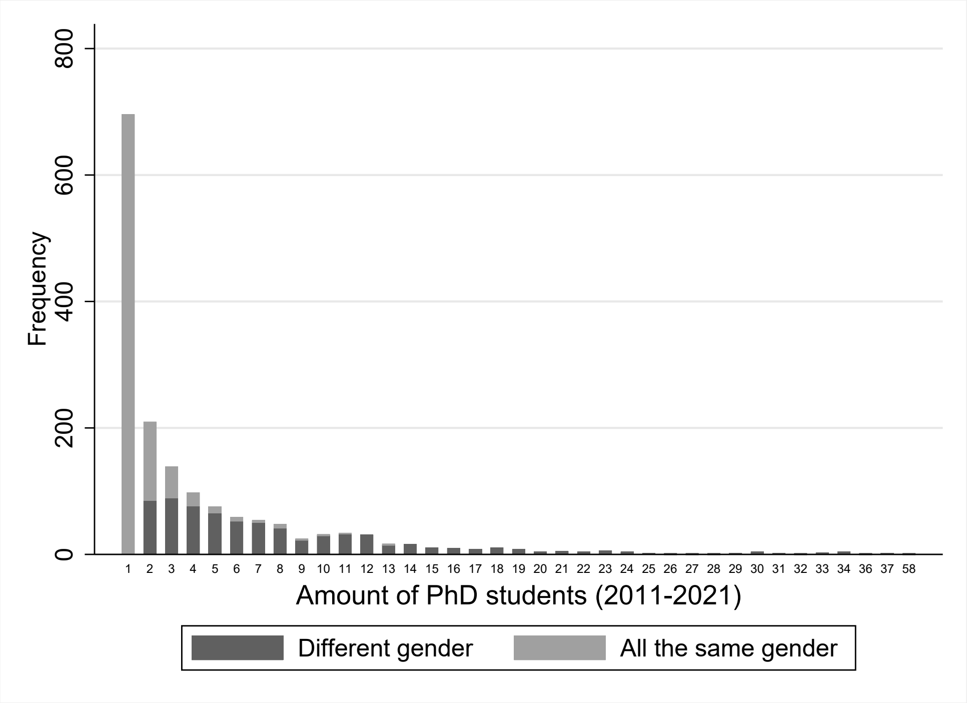

Figure S5 also shows whether the PhD students within one doctoral advisor had the same gender. Per definition, all doctoral advisors that finished supervising just one PhD dissertation belong to the group of doctoral advisors without gender variation among their supervisees. As the number of PhD students per supervisor increase, the likelihood of them all having the same gender decreases. Of the 1,628 doctoral

advisors in the data, 43% has supervised multiple PhD students that do not all have the same gender. It is this group over which we are able to estimate the fixed effects models.

A worry might be that this group is selective, as strong bias might already play out in the hiring of PhD students. For that reason we also estimated the same models without including the fixed effect for doctoral advisor. As becomes clear from Tables S3 and S4, the estimates are very stable between the models with and without the fixed effects for doctoral advisors, indicating that the findings do not depend on the sample or method that was used.

3 Results from regression models

The estimates that are presented in Figure 1 and 2 in the article are based on simple descriptive statistics, whereas the estimates visualized in Figures 3 and 4 are derived from regression models. In this section we will discuss the outcomes of these regression models and some alternative specifications.

Table S3: Regression analyses on obtaining a 'cum laude' distinction

	(1)	(2)	(3)
Female PhD student	-0.029**	-0.033**	-0.035**
	(-4.78)	(-5.26)	(-5.82)
Academic field (ref= Econ. & Bus.)			
Humanities		0.021	0.170
		(1.19)	(1.88)
Law		0.045*	0.215*
		(2.01)	(2.30)
Medicine		0.005	0.138
		(0.30)	(1.55)
Social Sciences		0.061**	0.165
		(3.64)	(1.85)
Science		0.006	0.084
		(0.37)	(1.03)
Constant	0.064**	0.049**	-0.053
	(5.63)	(2.69)	(-0.67)
Year dummies	Yes	Yes	Yes
Advisor FE	No	No	Yes
N	5,239	5,239	7,249

t statistics in parentheses
* $p < 0.05$, ** $p < 0.01$

3.1 Main effect of gender of PhD

Table S3 presents the estimates of the main effect of the the gender of PhD students on their probability to obtain 'cum laude'. In the linear probability model the coefficients can be interpreted as a marginal change in percentage points (*pp*). Model 1 only included the gender of the PhD student and year dummies, to take out any general trend in gender inequality in 'cum laude' (see Section 4.1. Here we found that there is a baseline difference of 2.9 percentage points (-0.029) to the disadvantage of female PhD students.

In Model 2 we tested the Simpsons paradox (Figure 3), by including dummy variables for academic field. As visualized in the article, the gender gap in 'cum laude' distinctions is not affected by the inclusion by field of study and even becomes slightly

larger (3.3 *pp*). We did find some differences in the likelihood for 'cum laude' across academic fields. It is significantly larger in Law and Social Sciences compared to the reference category (Economics and Business), something that also descriptive became clear from Figure 2 (left panel).

Model 3, finally, exploited the within-doctoral advisor variation discussed in Equation 3. Here we included a fixed effect for doctoral advisor, and the predicted effect can therefore be interpreted as the average gender gap within doctoral advisors. After including advisor fixed effects, the estimate of the gender gap in 'cum laude' distinctions remains stable at -0.035 (3.5 *pp*).

3.2 Interaction analyses with advisors and dissertation committee

The next set of analyses (Table S4 dealt with the extent to which the gender gap in 'cum laude' depended on the doctoral advisor and the dissertation committee, as visualized in Figure 4 in the main article. Models 1 and 2 in Table S4 show the results of a regression where we looked at the extent to which the gender gap in 'cum laude' distinctions differs across male and female doctoral advisors. Model 1 makes clear that there was no main effect of having a female doctoral advisor: the probability of receiving 'cum laude' was not significantly associated with the gender of the doctoral advisor, and the effect was close to zero (0.005, or 0.5*pp*). In Model 2 we added the interaction between the gender of the PhD student and the gender of the doctoral advisor. While the interaction effect was positive (0.013, or 1.3*pp*), it does not reach conventional levels of statistical significance ($p > 0.05$). As presented in Figure 4, the regression models provided no evidence for an association between the gender gap in 'cum laude' and the gender of the doctoral advisor.

Models 3-5 of Table S4 dealt with the role of the dissertation committee. Here we also added committee size as control variable, since it might be that doctoral advisors compose a relatively large committee when they believe the dissertation to be of excellent quality. Model 3 presents the main effects of the committee and the committee size. There are some differences in the likelihood of receiving 'cum laude'; the average probability to receive 'cum laude' was highest in all-male committees (the reference category). The likelihood of receiving a 'cum laude' was 1.7*pp* (-0.017) smaller in a committee with up to 25% female members. There also was a positive association between committee size and 'cum laude': for each extra committee member the regression model predicted a 1.7*pp* increase in the probability of receiving a 'cum laude' distinction. Since this model includes advisor fixed effects, it was an effect estimated within the same doctoral advisor.

Model 4 is presents the analyses that are visualized in the main article (Figure 4,

Table S4: Interaction analyses with advisor and committee

	(1)	(2)	(3)	(4)	(5)
Female PhD student	-0.034**	-0.036**	-0.033**	-0.058**	-0.059**
	(-4.93)	(-5.02)	(-5.37)	(-5.00)	(-5.47)
Female doctoral advisor	0.005	-0.003			
	(0.75)	(-0.26)			
Female PhD * Female advisor		0.013			
		(0.88)			
Committee composition (ref. 0% Female)					
1-25%			-0.017*	-0.026*	-0.026*
			(-2.17)	(-2.49)	(-2.15)
26-50%			-0.015	-0.039**	-0.030*
			(-1.69)	(-3.25)	(-2.15)
51-100%			-0.025	-0.046*	-0.047*
			(-1.94)	(-2.10)	(-2.00)
Committee size			0.017**	0.017**	0.015**
			(3.32)	(3.31)	(3.49)
Interaction w/ committee (ref. 0% Female)					
Female PhD * 1-25% Female committee				0.022	0.029*
				(1.42)	(2.01)
Female PhD * 26-50% Female committee				0.047**	0.045**
				(2.99)	(2.78)
Female PhD * 51-100% Female committee				0.039	0.056*
				(1.56)	(2.13)
Constant	0.046**	0.047**	-0.133	-0.119	-0.017
	(2.60)	(2.62)	(-1.56)	(-1.40)	(-0.60)
Year dummies	Yes	Yes	Yes	Yes	Yes
Academic field dummies	Yes	Yes	Yes	Yes	Yes
Clustered SEs	Yes	Yes	No	No	Yes
Advisor FE	No	No	Yes	Yes	No
N	7,249	7,249	7,219	7,219	5,215

t statistics in parentheses
* $p < 0.05$, ** $p < 0.01$

right panel). It is the fixed effects model with the interaction between committee composition and gender of the PhD student. Model 4 makes clear that there was a significant interaction between the gender composition of the committee and the gender gap in 'cum laude' distinctions. The gap was largest (0.058 or 5.8*pp*) when the dissertation was evaluated by an all male committee. Compared to the 5.8, the gap decreases by 2.2*pp* when a committee includes up to 25% females, whereas the predicted gender gap is reduced by respectively 4.7*pp* and 3.9*pp* when the committee has 26-50% or 51-100% female members. In these committees the gender gap still exists, but becomes much smaller. Model 4 shows that the difference to the reference category (0% female members) is only significant when 26-50% of the committee members are female, the marginal predicted effects (main effect + interaction effect) that are portrayed in Figure 4 (left panel) tell a different story. Here it becomes clear that whenever a committee consists of at least 25% females, the gender gap in 'cum laude' is not significantly different from 0.

Figure S6: Predicted probability of 'cum laude' by committee composition

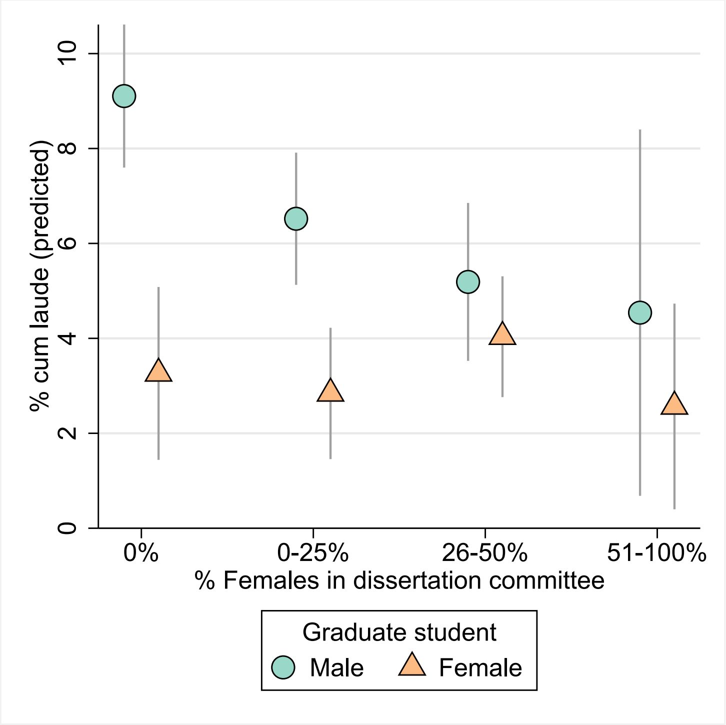

Figure S6 presents the same results as Figure 4 (right panel) as predicted probabilities for male and female PhD by the gender composition of the committee. Just like Figure 4 from main article, these results were based on Model 4 from Table S4. The figure makes clear that the larger gender gap in all-male committees was driven by a much higher share of male PhD students that received a 'cum laude' distinction. In all-male committees, a predicted 9.1% of male PhD students received 'cum laude', compared to 3.2% of female candidates–a difference by almost a factor 3. In line

with Figure 4 from the article, the gap decreases and becomes insignificant as the committee got closer to gender parity.

The estimates in Models 3 and 4 were obtained from a fixed effects model. The model has large advantages, as it controls for all unobserved variation between doctoral advisors. However, a disadvantage is that the results are based on a selective subgroup of doctoral advisors, namely those who had multiple PhD students in the observed period. Moreover, one could argue that part of the gender gap in 'cum laude' could be driven by bias in selection of PhD students, for example when male doctoral advisors are more likely to hire male PhD students.

For this reason, Model 5 investigated the effect of the committee composition, by using the sample of dissertations (N=5,215). In this model, both the within- and between-advisor effects were captured. The estimates in Model 5 are remarkably similar to those in Model 4. If anything, the results more strongly confirms an association between the gender composition of the dissertation committee and the gender gap in 'cum laude': the gender gap in committees where at least 50% of the members is female disappeared completely.

In a similar way to Figure 4 (left panel), the marginal effects obtained from Model 5 are displayed in Figure S7. This figure shows an even stronger association between committee composition and the gender gap in 'cum laude', supporting the conclusions drawn in the main article. Moreover, it indicates that the findings presented in the paper are robust to the method that was used.

Figure S7: Gender gap in 'cum laude' by committee (Model 5, Table S4)

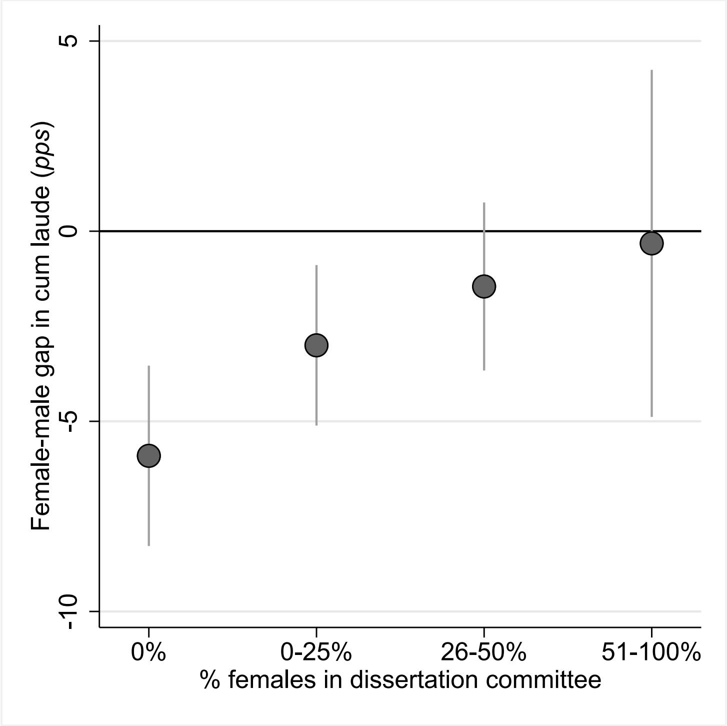

4 Robustness checks

4.1 Trend over time

In the article the gender gap in 'cum laude' distinctions was treated as static, even though the period under study spans over a decade (2011-2021). Earlier studies have argued that across several domains, gender inequality in science is decreasing, and gender bias is not as important of an explanation as it was some decades ago [2]. In order to investigate whether this argument also holds or our case, we have looked at whether the gender gap in 'cum laude' distinctions changed over time.

Table S5: Gender inequality in 'cum laude' over time

	(1)
Female PhD student	-0.040**
	(-3.33)
Year (0=2011)	-0.001
	(-0.91)
Female * Year	0.001
	(0.70)
Constant	0.057**
	(3.52)
Academic field dummies	Yes
N	5,239

t statistics in parentheses
* $p < 0.05$, ** $p < 0.01$

Table S5 presents the results of a regression analysis including an interaction between gender of the PhD student and a continuous variable for year (starting at 2011). The table makes clear that there was main effect of year, indicating that the probability of obtaining a 'cum laude' distinction had no linear association to the year of obtaining the doctoral degree. More importantly, the interaction effect of year of completion and female on obtaining 'cum laude' was not significant ($p > 0.05$) and not substantive in size: the predicted gender gap increased by 0.001 (or 0.1%) for every year.

It might be the case that the interaction with year is not linear but does change over time. To model this we interacted the gender of the PhD student with a set of dummy variables for year.[4] Figure S8 presents the trend over time, where the estimates were obtained from a regression where year (measured as a set of dummy variables) was interacted with the binary indicator for gender (1=female). The figure makes clear

[4]This is also the way in which year was controlled for in the other analyses: as a set of dummy variables to allow for non linear effects.

that there were some differences between the years in the observed period, but they do not follow a clear pattern, nor does it seem to be the case that the gender gap in 'cum laude' has decreased in the decade under study. In the regression model there are no significant differences between any of the year combinations ($p > 0.05$). In the case of 'cum laude' distinctions, we do not find evidence that it has decreased or even changed over time.

Figure S8: Gender gap in 'cum laude' by year

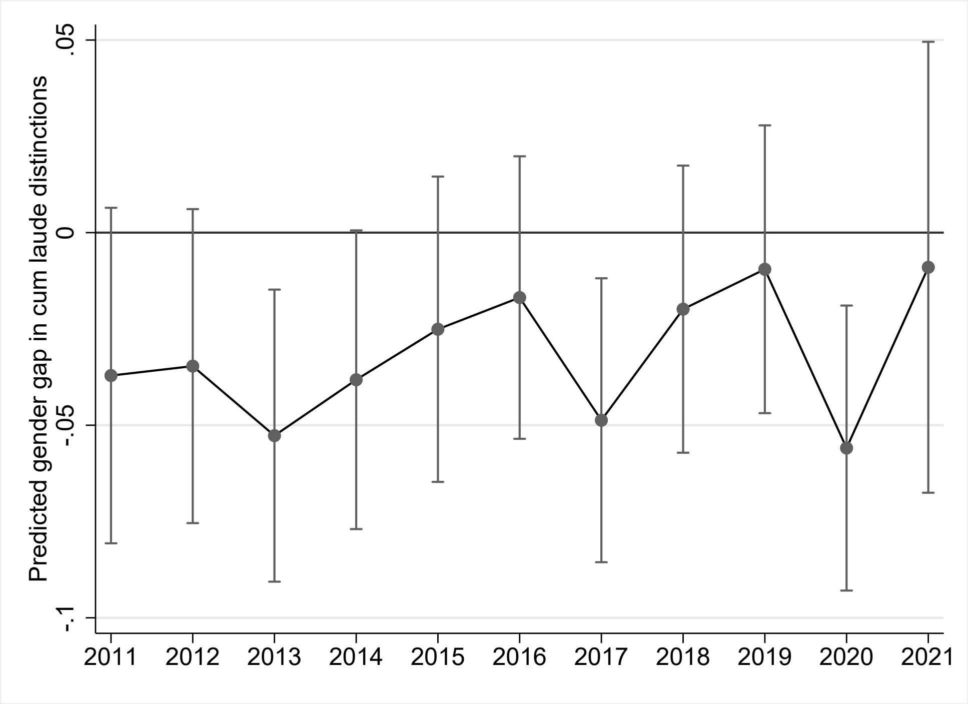

4.2 Role of co-supervisors

In Figure 4 (left panel) we have shown that the gender gap in 'cum laude' does not vary significantly between male and female doctoral advisors. As became clear from Section 1.1.1, almost all PhD students have co-supervisors next to their doctoral advisors. The co-supervisors were more likely to be female than the doctoral advisors: whereas only 19% of the doctoral advisors was female, 34% of all co-supervisors was female. It might be that the composition of the supervisory team does play a role, as here there will be more diversity than among the doctoral advisors.

For this reason we have re-estimated the analyses using the percentage of all supervisors that was female. For example, if someone had a male doctoral advisor, and two female co-supervisors, this percentage would be 67%. Figure S9a shows that the majority of supervisory teams consisted of only men: 53% of all PhD students had no female member in the supervisory team. Only 12% of the PhD students in the sample had a supervisory team where a majority of members was female.

Figure S9b shows whether there is an association between the composition of the supervisory team and the predicted gender gap in 'cum laude' distinctions. This figure is based on a regression model where a categorical variable of the gender composition of the supervisors (in three categories) was interacted with the gender of the PhD student (1=female). In line with what was found in Figure 4 (left panel), the effects are not significantly different across the three categories. The effect sizes are almost exactly the same when the supervisory team only consists of men or has at most 33% of female members. When more than one third of the members is female, the gender gap was smaller (about 1.7 times as small), but this difference is not significant ($p > 0.05$).

Figure S9: Composition of supervisors

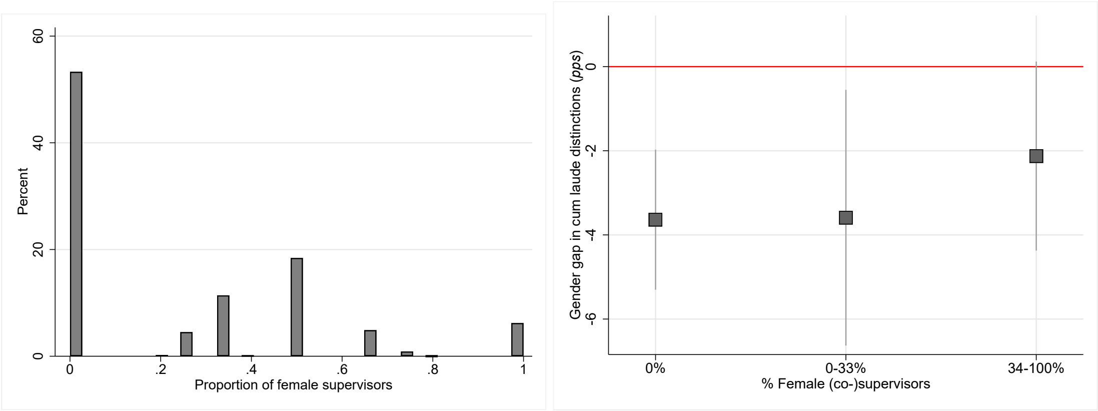

(a) Composition of supervisors

(b) Results from regression analyses

4.3 Seniority of committee

It might be that the likelihood to award a 'cum laude' distinction depends on the seniority of those who evaluate the dissertation. One explicit question in the 'cum laude' evaluation is whether the thesis belongs to the best theses that evaluator has read in a given field. It could be the case that this probability increases with seniority: as scholars have evaluated more theses, they are better able to judge whether the given thesis is "excellent". Given that women in committee are less likely to be senior, given that many more men are full professor in the Netherlands [1], it might confound the association found between the proportion of female committee members and the gender gap in 'cum laude'.

In order to investigate this, we looked at the composition of the dissertation committee, and more specifically at what proportion of the committee members was full professor at time time of evaluation. When the smaller gender gap in 'cum laude' in committee with more female members is confounded by their seniority, the current measure should provide an indication. Whether a given committee member is full professor or not is not registered for the full sample. Only from 2015 onward, this

information in available. This means that the analyses to answer this question were done on a smaller sample.

Table S6: Interaction analyses with advisor and committee

	(1)	(2)	(3)	(4)
Female PhD student	-0.057**	0.038	-0.064**	0.075
	(-3.47)	(0.77)	(-3.91)	(1.54)
Committee composition (ref. 0% Female)				
1-25%	-0.023	-0.022	-0.034*	-0.032*
	(-1.73)	(-1.62)	(-2.51)	(-2.33)
26-50%	-0.021	-0.019	-0.039*	-0.034*
	(-1.44)	(-1.24)	(-2.57)	(-2.24)
51-100%	-0.019	-0.014	-0.031	-0.021
	(-0.75)	(-0.52)	(-1.18)	(-0.80)
Interaction w/ committee (ref. 0% Female)				
Female PhD * 1-25% Female committee	0.032	0.031	0.036	0.034
	(1.53)	(1.48)	(1.73)	(1.67)
Female PhD * 26-50% Female committee	0.044*	0.041*	0.053*	0.049*
	(2.12)	(1.98)	(2.57)	(2.37)
Female PhD * 51-100% Female committee	0.033	0.026	0.031	0.020
	(1.05)	(0.85)	(1.03)	(0.67)
Committee size	0.016**	0.016**	0.020**	0.020**
	(2.65)	(2.78)	(2.93)	(2.98)
Professor composition committee		0.105*		0.146**
		(2.56)		(3.53)
Female PhD student * Professor composition		-0.111*		-0.162**
		(-2.04)		(-3.02)
Constant	-0.037	-0.133*	-0.039	-0.163
	(-0.94)	(-2.43)	(-0.27)	(-1.08)
Year dummies	Yes	Yes	Yes	Yes
Academic field dummies	Yes	Yes	Yes	Yes
Advisor FE	No	No	Yes	Yes
N	3,384	3,384	4,817	4,817

t statistics in parentheses
* $p < 0.05$, ** $p < 0.01$

Table S6 shows the results of these analyses. There are two different models again: models on the sample of students i (Models 1 and 2), and fixed effects models (Models 3 and 4). From Model 2 it becomes clear that the association between the gender gap in 'cum laude' and the gender composition of the dissertation committee is not affected by the inclusion of the interaction with the professor composition of the committee. The point estimates (0.032, 0.044, 0.033) remain largely similar, and the significance does not change. There is an independent (interaction) effect of the professor composition in the dissertation committee. Model 2 shows that a male PhD

student is more likely to obtain 'cum laude' in a committee with a higher proportion of full professors. These findings are then replicated in Models 3 and 4, which rely on the fixed effects approach. In conclusion, the seniority of the committee is associated with the likelihood of female PhD students to obtain a 'cum laude' distinction, but this does not affect the main finding about the role of the female committee.

4.4 Logistic regressions

A final robustness check addresses the method of estimation: linear probability models. A worry might be that the event we study (obtaining a cum laude) is relatively uncommon, and so a linear probability model might not fit the data very well. For this reason the key model was re-estimated using logistic regression. Figure S10 presents a replication of the first models estimating the main effects. Here it becomes clear that the findings are very similar (and sometimes even larger) than those estimated in linear probability models. Female PhD students have about 60% lower odds of obtaining 'cum laude' than male candidates, and this predicted difference increases over the models. It is important to note that the final model–the one with advisor fixed effects–is estimated over a different sample than the linear probability model. In a logit fixed effects model, only advisors can be included that have variation in the dependent variable; that have at least one PhD candidate that has a 'cum laude' distinction. This reduces that sample to about 2,490 compared to the similar model with a linear probability regression. Irrespective, the findings are qualitatively highly stable: even within the same advisor, men are much more likely to obtain cum laude than women.

Figure S10: Gender gap in 'cum laude' (logistic regression estimates)

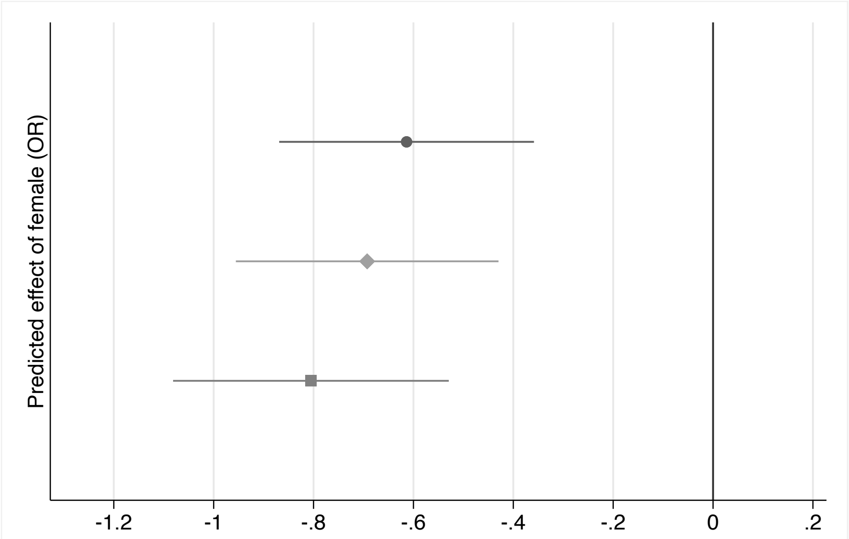

References

[1] LNVH, "Monitor Vrouwelijke Hoogleraren 2019," tech. rep., LNVH, Utrecht, 2020.

[2] S. J. Ceci and W. M. Williams, "Understanding current causes of women's underrepresentation in science," *Proceedings of the National Academy of Sciences*, vol. 108, no. 8, pp. 3157–3162, 2011.
